# Benralizumab in Corticosteroid-Refractory Drug Reaction With Eosinophilia and Systemic Symptoms: A Case Report

**DOI:** 10.7759/cureus.86932

**Published:** 2025-06-28

**Authors:** Katelin R Ross, Elijah Horesh, Milie M Fang, Jesse J Keller

**Affiliations:** 1 Dermatology, Oregon Health & Science University, Portland, USA

**Keywords:** dress syndrome, drug rash, drug reactions, hypersensitivity rash, hypersensitivity reaction, inpatient dermatology, skin eruption, systemic therapy

## Abstract

Drug reaction with eosinophilia and systemic symptoms (DRESS) is a severe hypersensitivity reaction involving multiple organ systems. While high-dose systemic corticosteroids are the first-line treatment, some cases are refractory, and effective second-line options are limited. We report a case of a 20-year-old male with lamotrigine-induced DRESS, presenting with rash, fever, eosinophilia, and fulminant hepatic failure unresponsive to corticosteroids. Following a single subcutaneous dose of benralizumab, an interleukin-5 (IL-5) receptor monoclonal antibody, the patient exhibited rapid clinical and laboratory improvement, narrowly avoiding liver transplantation. Benralizumab has demonstrated efficacy in corticosteroid-refractory DRESS cases, often achieving resolution with a single dose. This case supports the potential role of IL-5 axis blockade as a promising therapeutic strategy in managing severe DRESS.

## Introduction

Drug reaction with eosinophilia and systemic symptoms (DRESS) is a severe, delayed hypersensitivity reaction that occurs three to eight weeks after exposure to the offending medication. It is characterized by a morbilliform skin eruption, fever, eosinophilia, and multisystem involvement. Although the clinical presentation is often heterogeneous, the liver is the most commonly affected organ, with injury ranging from mild transaminitis to hepatic necrosis [1]. High-dose systemic glucocorticoids are the first-line treatment after discontinuing the culprit agent; however, despite treatment initiation, 3.8%-10% of cases result in mortality, primarily due to fulminant hepatic failure and liver necrosis [2]. Second-line therapeutic options are limited and often carry significant side effects. In this report, we describe a severe case of steroid-refractory DRESS with acute fulminant hepatic failure that responded remarkably to benralizumab, an interleukin-5 (IL-5) receptor monoclonal antibody, thus avoiding the need for liver transplantation. This case supports recent reports of benralizumab as an effective management strategy in severe DRESS [3]. Given the limited effective second-line therapeutic options for steroid-refractory DRESS, this novel case of rapid clinical improvement, which prevented the need for liver transplantation, addresses a current gap in the literature and adds to the growing evidence for the therapeutic potential of benralizumab in severe DRESS.

## Case presentation

**Figure 1:**
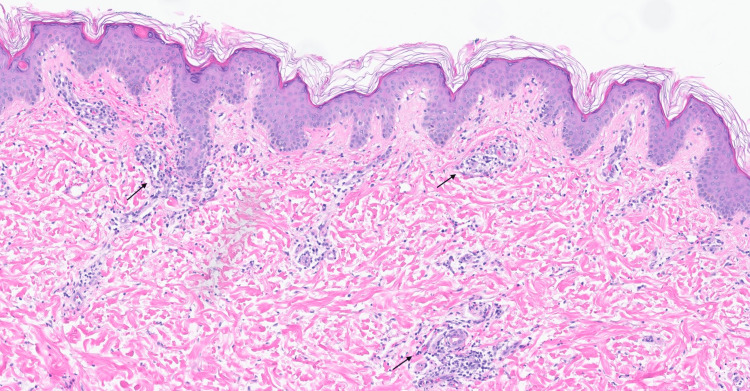
Histopathology from a skin biopsy of the right abdomen. Interface dermatitis with perivascular and interstitial lymphocytic infiltrate and rare neutrophils (arrows).

A 20-year-old male with epilepsy, on levetiracetam and tenofovir/emtricitabine for HIV pre-exposure prophylaxis (PrEP), developed a diffuse morbilliform rash, fever, lymphadenopathy, facial edema, and elevated liver enzymes. An extensive infectious workup, including blood cultures, urine studies, and tests for Group A Streptococcus, rubella, measles, cytomegalovirus, syphilis, Rocky Mountain spotted fever, hepatitis A, B, and C, HIV, HHV-6, Epstein-Barr virus, chlamydia, gonorrhea, and a comprehensive respiratory viral panel, returned negative results. The patient's history revealed that he had been started on lamotrigine three weeks prior. A skin biopsy showed interface dermatitis with perivascular and interstitial lymphocytic infiltrate and rare neutrophils (Figure 1). He was diagnosed with DRESS syndrome secondary to lamotrigine, with a Registry of Severe Cutaneous Adverse Reaction (RegiSCAR) score of 4, indicating probable DRESS. Lamotrigine was discontinued, and the patient was started on oral prednisone at 1 mg/kg/day. During his hospital stay, he experienced sharp chest pain. An electrocardiogram revealed findings consistent with acute pericarditis, and he was also started on colchicine. Following clinical improvement and normalization of liver enzymes over three days, he was discharged with a slow prednisone taper of 60 mg daily, reduced by 10 mg every seven days, with close outpatient follow-up.    

**Figure 2:**
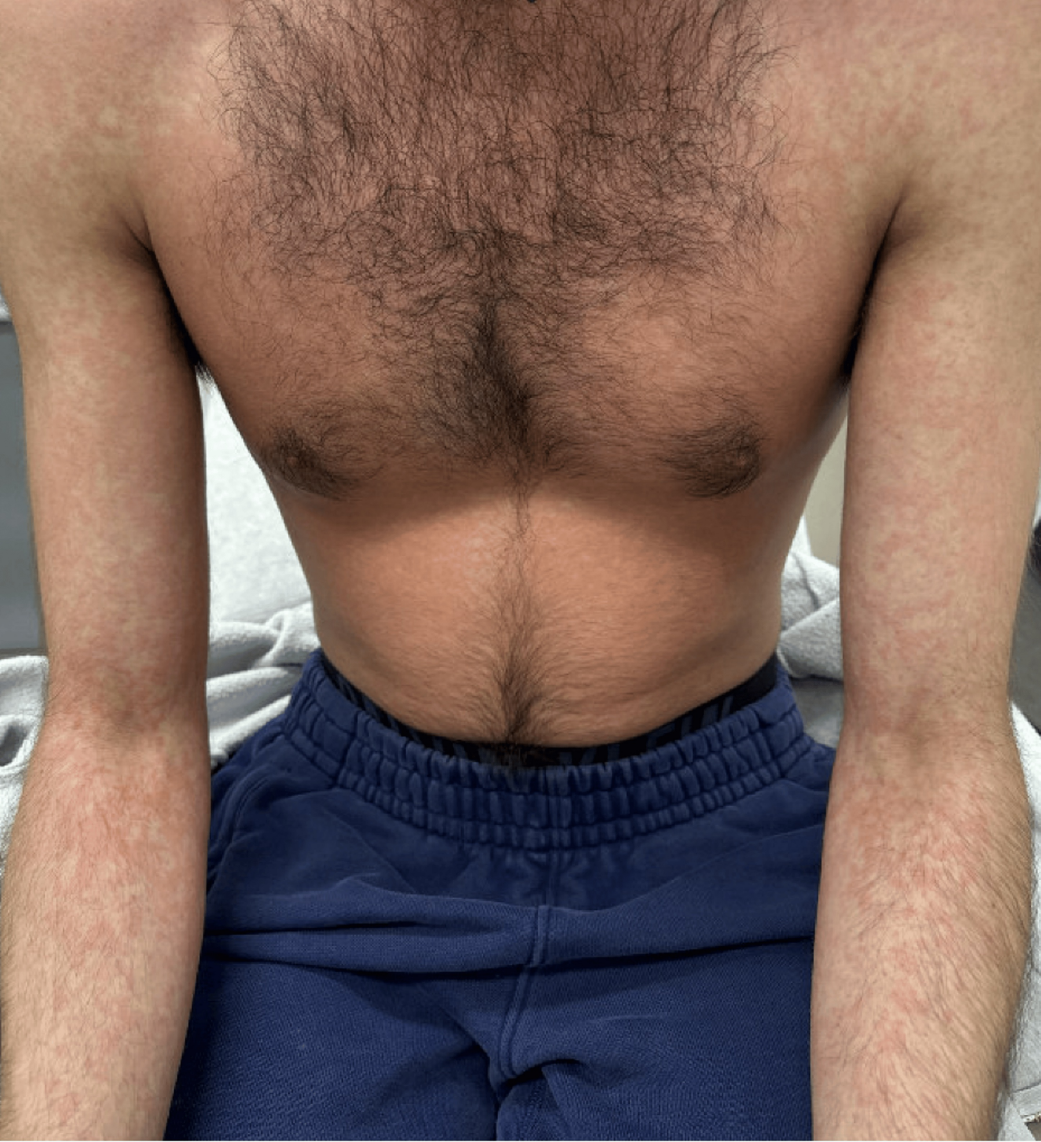
Morbilliform rash on anterior trunk and bilateral arms.

Five days after discharge, the patient was readmitted with a worsening morbilliform rash covering 85% of his body surface area, fevers, pharyngitis, eosinophilia, and lymphadenopathy (Figure 2). Although the patient had been on PrEP therapy for over seven months, making it an unlikely cause for his condition, the medication was discontinued, as tenofovir has been implicated in some cases of DRESS. The prednisone dosage was increased to 2 mg/kg/day without improvement. Laboratory values showed eosinophilia (2.61 × 10³/μL), leukocytosis (19.99 × 10³/μL), thrombocytopenia (88 x 10³/μL), and anemia (hemoglobin 12 g/dL). Within 72 hours of admission, he developed encephalopathy and rapidly rising liver enzymes (AST 7123 U/L, ALT 7897 U/L, and total bilirubin 18.1 mg/dL), along with elevated lactate at 4.2 mmol/L and an international normalized ratio (INR) of 2.52, raising concerns for acute fulminant hepatic failure. A request for a liver transplant was initiated, prompting additional evaluations including abdominal CT and ultrasound, expanded serologic testing to rule out other autoimmune and viral causes, and a recommendation for a percutaneous liver biopsy with copper quantification. The transplant hepatology team accepted the referral, and the abdominal transplant surgeon evaluated the patient and obtained consent in preparation for the liver transplant. 

The patient’s RegiSCAR score was 8, confirming a definitive case of severe DRESS. After input from multidisciplinary teams including dermatology, allergy and immunology, and primary care medicine, a single subcutaneous injection of benralizumab (30 mg) was administered. Subsequently, liver enzymes rapidly improved within days, encephalopathy resolved, eosinophil counts normalized, and dermatitis subsided. The liver transplant was canceled, and at a four-week follow-up, the patient demonstrated progressive clinical improvement with laboratory values within reference ranges. 

## Discussion

Managing refractory or relapsing DRESS syndrome presents significant challenges due to its heterogeneity and unpredictability, which can lead to potentially fatal consequences. The pathophysiology of DRESS involves the activation of T-lymphocytes that produce pro-inflammatory cytokines, including tumor necrosis factor-alpha (TNF-a), interferon gamma (IFN-γ), IL-5, and IL-6. Notably, IL-5 plays a critical role in eosinophil activation and chemotaxis. It is the most specific cytokine for eosinophil development, promoting maturation, mobilization, and prolonged survival, which contributes to the severe inflammation and organ damage characteristic of DRESS syndrome [1,4].

First-line therapy involves high-dose corticosteroids that are slowly tapered over several months; however, 10-15% of cases relapse despite treatment [5]. In this case, the patient’s initial response to corticosteroids was transient, highlighting the refractory nature of the disease and its evolving immunologic progression. This raises the possibility that early steroid responsiveness may diminish as the condition evolves from a CD8+ cytotoxic T-cell response to a CD4+ helper T-cell-mediated response, which is responsible for eosinophil-driven tissue injury. In this phase, corticosteroids may continue to suppress T-cell activity but fail to adequately suppress eosinophilic inflammation, allowing for continued eosinophil activation, tissue infiltration, and clinical relapse [6]. 

Second-line immunosuppressants, such as cyclosporine, cyclophosphamide, intravenous immunoglobulin, and Janus kinase inhibitors, have been used with varying degrees of success and are often associated with significant side effects. However, emerging evidence indicates that the IL-5 axis is a particularly promising therapeutic target [3]. Benralizumab, a biologic approved for treating severe eosinophilic asthma, is an IL-5 monoclonal antibody that binds to the IL-5 receptor alpha, which is present on eosinophils and basophils. Unlike other IL-5 axis therapies, benralizumab specifically targets the alpha subunit of the IL-5 receptor, inducing antibody-dependent cellular cytotoxicity, leading to rapid depletion of eosinophils in the bloodstream and tissues [4,7]. This mechanism suggests potential efficacy in treating other eosinophilic conditions, and its application has been reported in eosinophilic esophagitis, hypereosinophilic syndrome, and eosinophilic granulomatosis with polyangiitis, among others [8-10]. Utilizing this mechanism, it has also been employed as a therapeutic strategy for treating DRESS syndrome, providing a targeted approach where conventional treatments have failed.

Several case reports of steroid-refractory DRESS patients have been treated with IL-5 axis inhibitors, including benralizumab, mepolizumab, and reslizumab. Notably, nine cases were successfully treated with benralizumab (Table 1). While mepolizumab and reslizumab often required multiple doses over several months, benralizumab achieved clinical resolution with a single dose in most cases [3]. One patient experienced a relapse after four months but responded to subsequent mepolizumab therapy. Another patient had a fatal outcome attributed to COVID-19 complications. Corroborating these reports, our patient showed rapid clinical improvement after a single dose and remained symptom-free at follow-up. These findings suggest that IL-5 axis blockade, particularly with benralizumab, offers a promising, safe, steroid-sparing therapeutic option for managing severe, corticosteroid-refractory DRESS. 

**Table 1:** Published cases of DRESS treated with benralizumab. DRESS: drug reaction with eosinophilia and systemic symptoms; TEN: toxic epidermal necrolysis.

Publication (year)	Patient gender	Patient age	Suspected causative drug(s)	Initial therapy	Benralizumab dosage	Number of doses	Indication for benralizumab	Outcome
Schmid-Grendelmeier et al. (2021) [11]	Female	54	Esomeprazole and piperacillin	Methylprednisolone	30 mg	1	Steroid-resistant DRESS in the context of COVID-19	Clinical improvement
Schmid-Grendelmeier et al. (2021) [11]	Male	58	Midazolam	Methylprednisolone	30 mg	1	Steroid-resistant DRESS in the context of COVID-19	Clinical improvement; fatal outcome due to COVID-19 complications
Mesli et al. (2021) [12]	Male	43	Cefepime	Methylprednisolone, IVIG	30 mg	2	Steroid-resistant DRESS with hemophagocytic lymphohistiocytosis	Clinical improvement
Lang et al. (2021) [13]	Female	87	Allopurinol, pregabalin	Methylprednisolone	30 mg	1	Steroid-resistant DRESS	Clinical improvement
Lang et al. (2021) [13]	Male	74	Allopurinol	Methylprednisolone	30 mg	1	Steroid-resistant DRESS	Clinical improvement
Lang et al. (2021) [13]	Female	67	Ibuprofen, paracetamol	Methylprednisolone	30 mg	1	Steroid-resistant DRESS; relapse treated with 100 mg mepolizumab	Clinical improvement; relapse occurred after four weeks
Zeller et al. (2022) [14]	Male	75	Not specified	Methylprednisolone	30 mg	1	Steroid-resistant TEN/DRESS overlap	Clinical improvement
Pintea et al. (2022) [15]	Female	78	Allopurinol	Prednisone	30 mg	1	Steroid-resistant DRESS	Clinical improvement
Zhang and Wangberg (2024) [16]	Male	65	Bortezomib, Lenalidomide	Corticosteroids, Cyclosporine, Mepolizumab	Unspecified	N/A	Steroid-resistant DRESS; failed cyclosporine, mepolizumab	Clinical improvement

Theoretical risks of depleting eosinophils through IL-5 blockade include impaired host defense against certain infections, especially parasites and helminths, as well as potential roles in cancer defense [17]. In specific tissues like the gastrointestinal tract and lungs, resident eosinophils are important for maintaining local immune balance and barrier function, indicating a possible risk of disrupting tissue homeostasis when these cells are depleted [4]. However, eosinophils in tissues such as the lungs and duodenum often lack the IL-5 receptor and are therefore unaffected by IL-5-targeted therapies, and clinical experience has not yet shown a significant rise in infections [4]. Benralizumab has demonstrated a favorable safety profile, even with extended use in clinical trials for COPD and asthma, up to one and two years, respectively, with no notable increase in infections or malignancies [17,18].

Considering the relative safety of benralizumab compared to extended courses of systemic corticosteroids, earlier use of anti-IL-5 therapy or even replacing steroid treatment entirely in some patients could be a practical option for future DRESS management. Although currently used only off-label, benralizumab's limitations include access and cost, emphasizing the need for further research and validation of this potential treatment. Nonetheless, close monitoring of liver, kidney, and blood test results, along with any new signs of infection, remains essential in acute, multisystem conditions like DRESS syndrome.

## Conclusions

This case demonstrates the effective use of anti-IL-5 therapy in managing severe, corticosteroid-refractory DRESS syndrome with fulminant liver failure, thereby avoiding the need for an imminent liver transplant. These findings support the potential of IL-5 axis blockade as a promising approach. Further research, including the development of patient registries and prospective data collection, is necessary to confirm its effectiveness and establish standardized treatment protocols.
